# Zika E Glycan Loop Region and Guillain–Barré Syndrome-Related Proteins: A Possible Molecular Mimicry to Be Taken in Account for Vaccine Development

**DOI:** 10.3390/vaccines9030283

**Published:** 2021-03-19

**Authors:** Grégorie Lebeau, Etienne Frumence, Jonathan Turpin, Floran Begue, Jean-Jacques Hoarau, Gilles Gadea, Pascale Krejbich-Trotot, Philippe Desprès, Wildriss Viranaicken

**Affiliations:** 1 Processus Infectieux en Milieu Insulaire et Tropical (PIMIT), Université de La Réunion 1, La Réunion, 97490 Sainte-Clotilde, France; greg.lebeau@live.fr (G.L.); Etienne.frum@gmail.com (E.F.); jonathan.turpin@univ-reunion.fr (J.T.); jean-jacques.hoarau@univ-reunion.fr (J.-J.H.); gilles.gadea@inserm.fr (G.G.); pascale.krejbich@univ-reunion.fr (P.K.-T.); philippe.despres@univ-reunion.fr (P.D.); 2 Université de La Réunion, INSERM, UMR 1188 Diabète athérothombose Réunion Océan Indien (DéTROI), 97490 Saint-Clotilde, France; begue.floran@hotmail.fr

**Keywords:** ZIKV, Guillain–Barré syndrome, molecular mimicry, calcium channel voltage dependent, heat shock protein, vaccine

## Abstract

The neurological complications of infection by the mosquito-borne Zika virus (ZIKV) include Guillain–Barré syndrome (GBS), an acute inflammatory demyelinating polyneuritis. GBS was first associated with recent ZIKV epidemics caused by the emergence of the ZIKV Asian lineage in South Pacific. Here, we hypothesize that ZIKV-associated GBS relates to a molecular mimicry between viral envelope E (E) protein and neural proteins involved in GBS. The analysis of the ZIKV epidemic strains showed that the glycan loop (GL) region of the E protein includes an IVNDT motif which is conserved in voltage-dependent L-type calcium channel subunit alpha-1C (Ca_v_1.2) and Heat Shock 70 kDa protein 12A (HSP70 12A). Both VSCC-alpha 1C and HSP70 12A belong to protein families which have been associated with neurological autoimmune diseases in central nervous system. The purpose of our *in silico* analysis is to point out that IVNDT motif of ZIKV E-GL region should be taken in consideration for the development of safe and effective anti-Zika vaccines by precluding the possibility of adverse neurologic events including autoimmune diseases such as GBS through a potent mimicry with Heat Shock 70 kDa protein 12A (HSP70 12A).

## 1. Introduction

In the last few decades, there has been an increasing number of epidemics associated with mosquito-borne RNA viruses of medical concern such as Dengue Virus (DENV), West Nile Virus (WNV) or Chikungunya Virus [1]. Like DENV and WNV, Zika Virus (ZIKV) belongs to the flavivirus genus of the *Flaviviridae* family [2]. Classically, ZIKV is transmitted by mosquito bite and the preferred vectors for ZIKV spreading are *Aedes* genus mosquitoes [2,3]. ZIKV was first reported in Uganda in 1947 in a rhesus monkey; however, the virus remained silent for years, only provoking sporadic infections until 2007 [2]. The outbreak worldwide spread urged WHO to declare ZIKV as a major public health issue, leading to a global effort to fight against the disease. ZIKV infection causes clinical manifestation in ∼18% of cases ranging from mild disease with a denguelike syndrome, to more severe outcomes such as congenital microcephaly [4,5,6]. Of note, other neurological disorders have been associated with ZIKV infection, including encephalitis, myelitis and, finally, Guillain–Barré Syndrome (GBS) [5,6,7,8]. Moreover, uncommon modes of transmission have been described, including sexual and vertical (mother-to-infant) transmission, increasing the threat that this virus represents [9,10,11,12].

Like other flaviviruses, ZIKV is a positive sense single-stranded RNA virus. The viral genomic RNA encodes a large polyprotein, which is processed to generate the structural proteins C, prM and E followed by the nonstructural proteins NS1 to NS5. The E protein is involved in virus binding onto the host-cell and the internalization of the viral particles. The E ectodomain is divided into three domains: I (EDI), II (EDII), and III (EDIII). The EDI comprises a glycan loop which is glycosylated on N154. The antibody-mediated virus neutralization depends on the availability of neutralizing epitopes on the E protein.

To date, there are two ZIKV lineages: the African lineage with the historical strain MR766 (1947) as prototype and the Asian lineage, the latter being responsible for the contemporary epidemics in the South Pacific and the Americas. Critically, the Asian lineage is the only one associated with GBS so far. Comparative analysis between Asian lineage ZIKV strains PF13 (French Polynesia, 2013) and BeH819015 (Brazil, 2015) with African strain MR766 showed differences in their ability to infect human host cells [13]. The epidemic ZIKV strains differ from African strain MR766-NIID by eight amino acid substitutions in ZIKV E protein at positions E-152/156/158/169/285/341/343. Interestingly, the residues E-152/156/158 are identified into the glycan loop (GL, residues E-151 to E-165) region of ZIKV. The GL region of Asian lineage ZIKV strains has a glycan linked to N154 but the African strain MR766 does not. We previously reported that the GL region influences the availability of neutralizing epitopes on ZIKV [14] and that residues E-152/156/158 play a key role in the antigenic reactivity of the GL region [14]. In the present study, we proposed that amino acids at positions E-152/156/158 of the ZIKV epidemic strains might exhibit a molecular mimicry with GBS-related proteins. The possibility that E-clusters of residues E-283/285, E-341/343, and E-437/438 could account for a molecular mimicry in relation with human proteins was also investigated.

## 2. Materials and Methods

We conducted a comparative analysis between the E sequences from epidemic Brazilian ZIKV strain BeH-819015 (Genbank access number KU365778) isolated in 2015 and laboratory-adapted African ZIKV strain MR766-NIID lineage isolated in 1947 (Genbank access number LC002520). It is of note that biological properties of viral strains BeH-819015 and MR766-NIID have been extensively studied [13,14,15,16,17].

We attempted to determine potential candidates for Asian lineage ZIKV E protein molecular mimicry using Blastp suite (https://blast.ncbi.nlm.nih.gov/Blast.cgi?PAGE=Proteins, accessed on 11 November 2020) included in BLAST^®^ NCBI tool.

For this purpose, we entered as a query the diverse peptides resulting from substitutions only observed in the epidemic strains of ZIKV, limiting ourselves to the small size peptides (5 amino acids). Then, we searched for protein with these sequences conserved among human proteins (taxid: 9605) using the reference proteins database (refseq_protein) and protein–protein BLAST algorithm with default search parameters. Selection criteria for *in silico* determination of mimicry candidates among sequences that produced significant alignment were defined as follows: (i) primary sequence fully conserved in human candidate protein; (ii) candidate protein highly expressed in nervous system, both central and peripheral; (iii) candidate protein near or far associated with neuropathies, in the literature.

Additionally, the entire primary amino acid sequence of ZIKV (epidemic strain) envelope protein was assessed using B Cell Epitope Prediction Tools from the Immune Epitope Database (IEDB; http://tools.iedb.org/bcell/, accessed on 12 December 2020). Briefly, ZIKV E sequence was entered in the Antibody Epitope Prediction tool set on Bepipred Linear Epitope Prediction 2.0 in order to determine potential epitopes leading to antibody response in E protein. Subsequently, these results were cross-checked with the query sequence previously used for Blastp analysis.

Finally, prediction of peptide folding was done using PEP-FOLD3 tool [18] (available at https://bioserv.rpbs.univ-paris-diderot.fr/services/PEP-FOLD3/, accessed on 3 March 2021). To do so, we entered 30 amino acids sequence containing the pentapeptide of interest (potentially involved in molecular mimicry), running prediction with 100 independent simulations and sorting models by sOPEP. Following sequences were used for prediction (in brackets, protein name—accession number): IMLSVHGSQHSGMIVNDTGHETDENRAKVEV (Zika Virus polyprotein, KU365778), FSPNNRFRLQCHRIVNDTIFTNLILFFILLS (Voltage-dependent L-type calcium channel subunit alpha-1C, Q13936) and GDTGITPLSPSHIVNDTDSNVSEQQSFLVV (Heat shock 70 kDa protein 12A, O43301).

**Figure 1:**
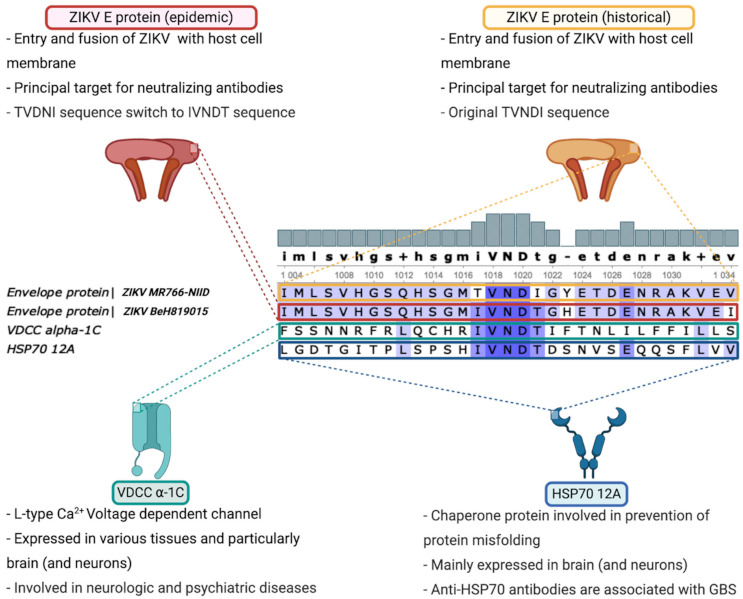
Calcium channel voltage-dependent L type α-1C subunit and Heat Shock 70 kDa protein 12A are potential candidates for molecular mimicry following ZIKV infection. Comparative analysis of Brazilian strain of ZIKV (BeH819015) and laboratory-adapted historical strain of ZIKV (MR766-NIID) revealed that an IVNDT polypeptide, only found in epidemic strain, might be related to ZIKV-related GBS due to calcium channel voltage-dependent L type α-1C subunit or Heat Shock 70 kDa protein 12A molecular mimicry.

The illustrations in figures 1 and 3 were made with the BioRender software.

## 3. Results

One of the potential mechanisms through which ZIKV is supposed to induce GBS is molecular mimicry [19,20]. This mechanism corresponds to a similar structure between pathogens and human proteins [21]. To explore this hypothesis, we decided to develop an *in silico* approach to determine whether ZIKV E protein shares homology with human proteins (Table 1). Thus far, GBS has been associated with contemporary epidemic ZIKV strains of Asian lineage. It seems unlikely that African lineage ZIKV have the ability to mediate GBS since no case was reported to date, which could be an artefact of a poor history of infections.

Based on the amino acid changes observed between the E proteins of viral strains BeH-819015 and MR766-NIID, we identified four clusters of amino acid substitutions making good candidates to further explore molecular mimicry hypothesis (Table 1). Into the EDI domain, residues at positions E-152/156/158 may play a key role in antigenic reactivity of ZIKV GL region [14]. The E-GL region of Asian lineage ZIKV encompasses the sequence IVNDT (residues E-152 to E-156) which includes the sequon NDT where a glycan is linked to N154. African ZIKV strain MR766-NIID includes the pentapeptide TVNDI in place of IVNDT due to the (T, I) permutation at positions E-152/156 leading to a nonglycosylated ZIKV [14]. The amino acid substitution E-Y158H has been identified between MR766-NIID and epidemic Brazilian strain BeH819015 of Asian lineage. It is of note that two substitutions at positions E-156/158 of the GL region change the pentapeptide DIGYE into DTGHE. Other amino acid residues in the E protein are candidates for searching for a sequence homology with GBS-related proteins. There are the EDII residues E-283/285 with a pentapeptide GKFLS for BeH819015 but GRLSS in MR766-NIID. Additionally, the EDIII residues E-341/343 compose the pentapeptide VPAQM in BeH819015 but IPVQM in MR766-NIID. Finally, the V437A and F438L permutations lead to a GALNS peptide for BeH819015 compared to GVFNS for MR766-NIID.

With the aim to understand the underlying mechanism for ZIKV-mediated GBS, *in silico* methods were employed to identify potential protein candidates for molecular mimicry. Consequently, protein sequence alignment based on the E proteins of BeH819015 and MR766-NIID was assessed with human proteins, following the criteria described in the Materials and Methods section. From all the candidates found using this method (Table 1), calcium channel voltage-dependent L type, alpha 1C subunit and Heat Shock 70 kDa protein 12A were the only ones to fulfill all the criteria (Figure 1). Indeed, the IVNDT sequence is totally conserved and these proteins are highly expressed in the nervous system, both central and peripheral [22,23,24]. Likewise, as described below, a correlation has been reported between development of neuropathies and autoantibodies directed against both the voltage-dependent Ca^2+^ channel and HSP70. Thus, these proteins could be exposed to the immune system and therefore to an autoantibody response.

Moreover, according to antibody epitope prediction using IEDB tools, we determined that among all the predicted epitopes potentially leading to antibody response in ZIKV E sequence, only two peptides relate to regions where amino acid changes were observed in the epidemic ZIKV strain and that served in the previously cited alignment (Table 2). Strikingly, one of them contained the IVNDT sequence. Considering that other predicted antibody epitopes do not include any known amino acid substitutions between African- and Asian lineage ZIKV, it is highly unlikely that such E-associated epitopes may play a role in the development of ZIKV-mediated GBS. However, in order to rule out any possibility, we tested all the pentapeptides derived from each of these epitopes, using the methodology described previously. Conversely to IVDNT previously mentioned, no matching candidate was found with any of the tested pentapeptides here (Tables S1–S3).

In addition, a previous work has shown that susceptibility to Zika virus infection is increased in differentiated neural cells compared to their undifferentiated counterparts [25]. Additionally, this work suggests that this may be related to the several neurological outcomes of ZIKV infection [25]. Thus, we wondered if one or several GBS-related proteins could be differentially expressed in differentiated neural cells compared to undifferentiated neural cells, maybe explaining the development of GBS following ZIKV infection. To answer this, we performed a differential expression analysis of the transcriptome dataset GSE121534 using DEBrowser [26] as described in Supplemental Materials. We compared a list of genes with a fold value >5 (Table S1) to a set of GBS-related proteins summarized in Table S2, but no match was observed between the transcriptome dataset GSE121534 and the list of GBS-related proteins. Remarkably, calcium channel voltage-dependent L type, alpha 1C subunit and Heat Shock 70 kDa protein 12A were expressed by differentiated and undifferentiated SH-SY5Y in the transcriptome dataset GSE121534 analysis. This led us, again, to privilege the hypothesis of molecular mimicry with these two proteins.

**Figure 2:**
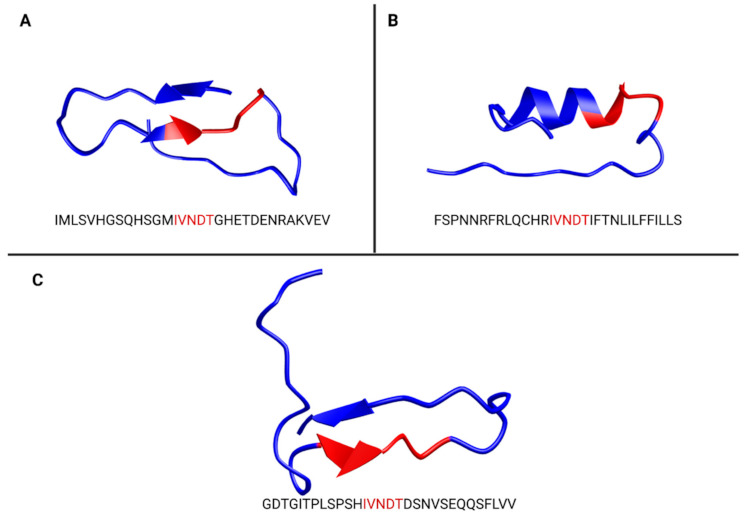
Predicted folding of epitope containing IVNDT (in red). ZIKV Asian lineage (**A**), VDDC alpha 1C (**B**) and HSP70 12 A (**C**). Prediction of peptide folding was done using PEP-FOLD3. HSP70 12A folding seems to be close to ZIKV E glycan loop folding, conversely to VDDC alpha 1C.

Finally, based on the predicted secondary structure data (Figure 2) obtained using PEP-FOLD3 [18], as described in the Materials and Methods section, it seems that the folding of the ZIKV E glycan loop from the Asian lineage is close to Heat Shock 70 kDa protein 12A. Indeed, in both cases, IVNDT sequence belongs to a β-sheet structure, strengthening the theory that this pentapeptide could elicit an autoimmune response. Taken altogether, these data form a growing body of evidence for antibody-directed against IVNDT peptide and either calcium channel voltage-dependent L type, alpha 1C subunit or Heat Shock 70 kDa protein 12A.

## 4. Discussion

**Figure 3:**
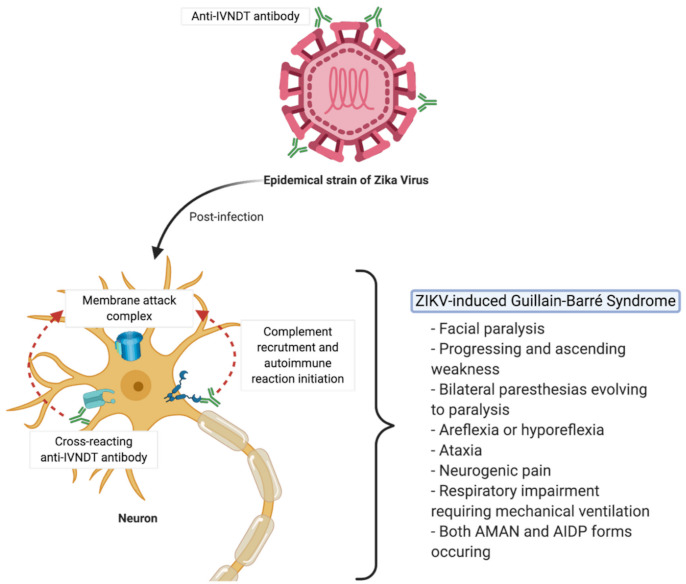
ZIKV-induced Guillain–Barré Syndrome might be promoted by autoantibodies directed against calcium channel voltage-dependent L type α-1C subunit or Heat Shock 70 kDa protein 12A.

Voltage-dependent calcium channels (VDCCs) are key proteins modulating Ca^2+^ entry into electrically excitable cells, following depolarization. Calcium channel voltage-dependent L type α-1C subunit, also called Ca_v_1.2, is part of L-type VDCCs family [22]. This Ca^2+^ channel participates in hippocampal long-term potentiation, hippocampus-dependent forms of memory, peripheral vascular resistance, cardiac inotropy or insulin secretion [22,27]. The impairment of similar channels by anti-GM1 antibodies was implicated in development of GBS [28,29]. Since anti-GM1 antibodies are found at low levels in ZIKV-induced GBS patients, it is likely that voltage-dependent Ca^2+^ channel is targeted by different cross-reacting antibodies. Here, we showed the existence of an IVNDT conserved sequence between Ca_v_1.2 and ZIKV (BEH819015) envelope protein. Even if Ca_v_1.2 has never been directly implicated into development of GBS, nor any other neuropathy, we suggest that the recognition of Ca_v_1.2 on a neuron by cross-reacting anti-IVNDT antibodies following ZIKV infection might lead to the initiation of the immune response and development of the GBS pathophysiology (Figure 3). However, this hypothesis remains to be tested. Since Ca_v_1.2 is mainly involved in cardiac and memory functions, the development of the symptoms associated with these functions should be expected (e.g., cardiac and psychiatric forms), but to date none was reported. Moreover, Cav1.2 folding in the region of the IVNDT pentapeptide seems to be quite far from ZIKV E glycan loop folding (Figure 2A,B). Of note, predicting Ca_v_1.2 topology using PredictProtein [30], we noticed that IVNDT region is likely to be cytoplasmic, decreasing the probability of molecular mimicry with this candidate.

Heat Shock Proteins (HSP) are divided into subfamilies according to their theoretical molecular weights (27-kDa, 60-kDa, 70-kDa and 90-kDa). They are known for their crucial role in preventing protein misfolding and aggregation, as for their induction in case of cellular stress such as increased temperature radiation, exposure to chemicals, oxidative stress and various physiological and pathological stimuli [31]. An additional role of HSP is the participation in antigen presentation/cross-presentation [32] and inflammatory signaling [33]. Among this large family, HSP70s (to which belongs Heat Shock 70-kDa protein 12A—HSP70 12A) have been associated with autoimmune neurological disorders and more specifically immune-induced neuropathies like GBS [34]. Indeed, an increased prevalence of anti-HSP70 antibodies in GBS patients has been reported [31,35]. Here, we showed the existence of an IVNDT conserved sequence between HSP70 12A and ZIKV (BeH819015) envelope protein. As HSP70 12A is mainly expressed in neural cells of the central nervous system (CNS), such a finding might be meaningful for ZIKV-associated GBS pathophysiology insights. Additionally, in contrast to Cav1.2, HSP70 12A folding obtained via PEP-FOLD3 [18] fits well with the ZIKV E glycan loop from the Asian lineage (Figure 2A,C). Accordingly, the body of evidence would be more directed towards molecular mimicry against HSP70 12A. Based on our molecular mimicry hypothesis, it is proposed that anti-IVNDT antibodies produced during ZIKV infection cross-react with HSP70 expressed by neurons promoting complement recruitment, membrane attack complex formation and autoimmune reaction initiation (Figure 3).

Our *in silico* data allowed us to propose a molecular mimicry hypothesis between the Zika E sequence IVNDT and human neuronal proteins (e.g., Ca_v_1.2 and HSP70 12A) within the CNS. The pentapeptide IVNDT which composes the sequon NDT into the EDI domain of Asian lineage ZIKV is highly conserved among contemporary epidemic viral strains. Different substrains of the historical African strain MR766 as a ZIKV prototype have been identified as nonglycosylated ZIKV. Thus, MR766 substrain NIID (Genbank access number LC002520.1) contains a TVNDI sequence in the glycan loop region, whereas M.mulatta-tc/UGA/1947/MR-766 substrain (Genbank access number KU955594) bears a 4-aa deletion leading to a lack of VNDI sequence. To our knowledge, nonglycosylated ZIKV strains have never been involved in ZIKV-mediated GBS. It is therefore tempting to propose a link between the pentapeptide IVNDT found in Asian lineage ZIKV in relation with human proteins Ca_v_1.2 and HSP70 12A and the development of GBS in Zika patients. Our molecular mimicry hypothesis will need to be proven with the detection of anti-IVNDT cross-reacting antibodies in GBS patients diagnosed for ZIKV infection as well as the development of an animal model for understanding the mechanisms by which IVNDT peptide-related antibodies could trigger GBS.

In conclusion, we propose that Zika IVNDT peptide should be taken in consideration to preclude the risks of adverse neurologic autoimmune diseases such as GBS during the course of ZIKV infection. Our molecular mimicry hypothesis raises the question of vaccine development against ZIKV. Indeed, a possible causal relationship between molecular mimicry and the development of autoimmunity in response to ZIKV infection alerts us to the need for vaccine candidates exempt from the viral antibody epitopes identified as potential GBS triggers. We recently reported the development of a chimeric viral clone called ZIKALIVax, which was designed with viral strain MR766-NIID as backbone and structural protein region of epidemic strain BeH918015 [14]. The replacement of pentapeptide IVNDT into the BeH918015 glycan loop region by the TVNDI sequence from MR766-NIID resulted in a nonglycosylated envelope protein [14]. ZIKALIVax was proposed as a live-attenuated vaccine candidate against ZIKV-related disease [14]. Based on our molecular mimicry hypothesis between ZIKV sequence IVNDT and human proteins Ca_v_1.2 and HSP70 12A, it is presumed that ZIKALIVax cannot trigger GBS upon vaccination, reinforcing the attractivity of a such chimeric viral clone as a vaccine candidate against Zika.

## Figures and Tables

**Table 1:** Host proteins obtained from query sequence alignment using Blastp. The proteins highlighted in **bold** are the only ones which fulfill the criteria of selection as candidates for molecular mimicry.

Query Sequence	Substitutions Associated	Output
IVNDT	T152I/I156T	**Calcium channel voltage-dependent L type α-1C subunit** **Heat Shock 70 kDa protein 12A** Pecanexlike protein 2Cyclin-C Adhesion G-protein coupled receptor V1 Coagulation factor VIII HEAT repeat-containing protein 5B Sodium leak channel nonselective protein Hexokinase-1, -2, -3, HKDC1
DTGHE	I156T/Y158H	Macrophage colony-stimulating factor 1 Collagen and calcium-binding EGF domain-containing protein 1 Transcription factor COE1 Cytoplasmic FMR1-interacting protein 1 A disintegrin and metalloproteinase with thrombospondin motifs 2 Folliculin-interacting protein 1 Ubiquitin-associated protein 2-like Prolyl endopeptidaselike Adapter protein CIKS Dermokine Immunity-related GTPase family M protein Tyrosine-protein kinase ABL2 Zinc finger protein 491 Obscurin Protein prune homolog 2 Otogelin eIF-2-alpha kinase activator GCN1 BAH and coiled-coil domain-containing protein 1 Centrosome-associated protein CEP250 Supervillin Ankyrin-1
GRLSS	K283R/F285S	Pikachurin precursor Hamartin Ankyrin repeat and LEM domain-containing protein 2 Testis-expressed protein 10 GRB2-associated and regulator of MAPK protein 2 Low-density lipoprotein receptor-related protein 3 Ligand of Numb protein X 2 Synaptotagminlike protein 4 Chromodomain Y-like protein 2 Oxygen-dependent coproporphyrinogen-III oxidase, mitochondrial precursor Sodium- and chloride-dependent GABA transporter 2 Acetyl-CoA acetyltransferase, cytosolic ER membrane protein complex subunit 10 Histamine H4 receptor Protein GOLM2 Protein FAM83A Uncharacterized protein C9orf163 Uncharacterized protein C3orf18 Mucin-16 Obscurin Nesprin-1 1-acyl-sn-glycerol-3-phosphate acyltransferase gamma E3 ubiquitin-protein ligase HERC2 Histone-lysine N-methyltransferase 2A Serine/threonine-protein kinase mTOR Myosin light chain kinase, smooth muscle
VPAQM	I341V/V343A	Zinc finger protein 646, 292, 831, GLI1 Galactoside alpha-(1,2)-fucosyltransferase 2 Tensin-2 Neurolysin, mitochondrial Filamin-A Spectrin beta chain, nonerythrocytic 5 Structure-specific endonuclease subunit SLX4 Adhesion G protein-coupled receptor L3 Tau-tubulin kinase 1 IQ domain-containing protein N N-acetylglucosamine-1-phosphotransferase subunits alpha/beta FERM domain-containing protein 4A RE1-silencing transcription factor Protocadherin-8 Protein transport protein Sec24D Adhesion G-protein coupled receptor G2 Protein FAN Kinesinlike protein KIF18B
GALNS	V437A/F438L	A-kinase anchor protein 12 EMILIN-1 precursor DENN domain-containing protein 1C Gelsolin Tudor domain-containing protein 10 CXXC-type zinc finger protein 4 Obscurin Microtubule-actin cross-linking factor 1 Usherin Ryanodine receptor 2 Dynein heavy chain 8, heavy chain 14, heavy chain 7 Sacsin Basement membrane-specific heparan sulfate proteoglycan core protein DNA-dependent protein kinase catalytic subunit Protein bassoon Transformation/transcription domain-associated protein Xin actin-binding repeat-containing protein 2 Protocadherin-16 precursor

**Table 2:** Antibody epitope prediction for epidemical ZIKV (BEH819015) envelope protein. Summary of all antibody predicted epitope using IEDB tools with ZIKV (BEH819015) envelope protein as query. In **bold** are highlighted the sequences with substitutions in BEH819015 strain compared with MR766-NIID strain.

Start-End	Peptide
5–9	GVSNR
66–103	SDMASDSRCPTQGEAYLDKQSDTQYVCKRTLVDRGWGN
126–133	TGKSIQPE
146–163	SQHSGM**IVNDT**GHETDEN
193–197	RTGLD
218–240	FHDIPLPWHAGADTGTPHWNNKE
274–279	EAEMDG
312–322	TFTKIPAETL
349–352	MQTL
368–371	STEN
395–408	KITHHWHRSGSTIG
428–440	AWDFGSVG**GALNS**

## Supplementary Materials

The following are available online at https://www.mdpi.com/2076-393X/9/3/283/s1, Supplemental methods, Table S1: Guillain Barré-related proteins; Table S2: Gene differentially expressed by differentiated versus undifferentiated SH-SY5Y; Table S3: Screening of pentapeptides derived from Table 2 epitope.

## Patent

Part of this work has been described in the patent entitled “Vaccine compositions comprising an attenuated mutant zika virus” under the number WO2017220748A1 (priority date 2016-06-23).

## Abbreviations

BeH819015	Clinical isolate of ZIKV collected in Brazil in 2015
Ca_v_1.2	Calcium channel voltage-dependent L-type α-1C subunit
GBS	Guillain–Barré Syndrome
HSP70 12A	Heat Shock 70 kDa protein 12A
MR766-NIID	Laboratory-adapted ZIKV strain isolated in Uganda in 1947
VDCC	Voltage-dependent calcium channel
ZIKV	Zika Virus
