# Supplementary material for: Zika E Glycan Loop Region and Guillain–Barré Syndrome-Related Proteins: A Possible Molecular Mimicry to Be Taken in Account for Vaccine Development
Source: Vaccines (Basel). 2021 Mar 19;9(3):283. doi: 10.3390/vaccines9030283 (PMC8003386; doi:10.3390/vaccines9030283)
Supplement: Supplementary file 1 [file vaccines-09-00283-s001.zip › Supplementary material vaccines-1116910_GL 04.03.2021.docx]

Supplementary materials

1. Supplementary methods
   1. Transcriptome differential expression analysis

We conducted a differential expression analysis of transcriptome GSE121534 (available at <https://www.ncbi.nlm.nih.gov/geo/query/acc.cgi?acc=GSE121534>) generated by Sánchez-San Martín et al. using undifferentiated and differentiated SH-SY5Y [18] . To do so, we used DEBrowser tool [19] (available at <https://debrowser.umassmed.edu/>). Briefly, data were prepared using low count filtering method set to max and then analyzed, comparing undifferentiated versus differentiated SH-SY5Y, with following parameters: DE method – DESeq2, Fit type – parametric, betaPrior – false, Test type – LRT and Shrinkage – none. Subsequently, the list of genes with a fold change value > 5 (up or down) was exported in Excel format for further analyses (**Table S1**). Indeed, the results were then compared to a list of genes encoding Guillain Barré Syndrome-related proteins (**Table S2**), retrieved using: guillain barre, miller fisher, demyelinating, polyneuropathy, axonal neuropathy, motor neuropathy, immune neuropathy as query UniprotKB database (<https://www.uniprot.org/>), limiting the results to human proteins. Also, thanks to literature reviewing, we had to **Table S2** some proteins known in literature to be linked to Guillain Barré Syndrome.

1. Supplementary materials

**Table S1. Gene differentially expressed by differentiated versus undifferentiated SH-SY5Y**

Available on associated Excel file.

List of the 64 genes differentially expressed between undifferentiated and differentiated SH-SY5Y with a fold change value >5. To generate this list, we analyzed the transcriptome GSE121534 with DEBrowser as described in *Supplemental methods.*

**Table S2. Guillain Barré-related proteins**

Available on associated Excel file.

Top – List of the 140 human proteins related to Guillain Barré and neuropathies, retrieved using guillain barre, miller fisher, demyelinating, polyneuropathy, axonal neuropathy, motor neuropathy and immune neuropathy as query in Uniprot database and limiting the results to human proteins. Bottom – Review of literature of human proteins related to neuropathies due to autoantibodies-directed response.

**Table S3. Screening of pentapeptides derived from Table 2 epitope**

Available on associated Excel file.

Using Blast tool as previously described in *Materials and methods*, we screened all the pentapeptides derived from the IEDB epitope mapping (**Table 2**), to rule out any molecular mimicry with Guillain Barré-related proteins in these sequences and confirm the specificity of the result obtained for IVNDT sequon.
